# Considerations of Pool Dimensions in the Forced Swim Test in Predicting the Potential Antidepressant Activity of Drugs

**DOI:** 10.3389/fnbeh.2021.757348

**Published:** 2022-01-07

**Authors:** Gilberto Uriel Rosas-Sánchez, León Jesús German-Ponciano, Juan Francisco Rodríguez-Landa

**Affiliations:** ^1^Programa de Doctorado en Neuroetología, Instituto de Neuroetología, Universidad Veracruzana, Xalapa, Mexico; ^2^Laboratorio de Neurofarmacología, Instituto de Neuroetología, Universidad Veracruzana, Xalapa, Mexico

**Keywords:** antidepressant activity, behavior, forced swim test pool, immobility behavior, psychopharmacology

## Introduction

The forced swim test (FST) was proposed by Porsolt et al. (1977a,b) as a relatively rapid test to identify new compounds with potential antidepressant activity in rats and mice. In this test, the total time of immobility (TTI) was proposed as a variable that reflects despair-like behavior, which could be reduced by clinically effective antidepressants (Porsolt et al., 1977a, 1978). Since then, diverse interpretations of immobility in the FST have arisen, including behavioral despair, learned helplessness, and depression-like behavior, even considering the FST as an animal model of depression (Porsolt, 2000; Cryan and Mombereau, 2004). Immobility was interpreted as a passive coping strategy, anxiety, and psychomotor retardation or as a behavior that is related to autism spectrum disorder (Anyan and Amir, 2018; Unal and Canbeyli, 2019). A reduction of immobility in the FST by drugs was interpreted as an antidepressant-like effect because clinically effective antidepressant drugs significantly reduce this behavior (Dalvi and Lucki, 1999; Porsolt, 2000). Important discussions of the meaning and interpretation of immobility in the FST in mice and rats have occurred (Molendijk and de Kloet, 2019, 2021), questioning whether the FST should or should not be considered an experimental model of depression that reproduces in rodents the large spectrum of complex signs and symptoms that characterize clinical depression (Molendijk and de Kloet, 2021). Additionally, discussions about ethical issues related with the FST have arisen questioning its use in the preclinical research of antidepressant drugs (Reardon, 2019; Trunnell and Carvalho, 2021). The relevance of this kind of discussion highlights the following: “*…we must critically assess how we are interpreting complex animal behavior to ensure we are not overly interpreting behaviors or potential missing out on alternative and fruitful direction*” (Gorman-Sandler and Hollis, 2021).

The present opinion article discusses how pool dimensions, among other factors, can influence the detection of immobility in the FST and how these dimensions may permit or prevent the detection of an anti-immobility effect of relatively low doses of antidepressant drugs.

## The Forced Swim Test

The FST is one of the most used behavioral tests in preclinical research to evaluate substances with potential antidepressant activity in mice and rats (Yankelevitch-Yahav et al., 2015; Anyan and Amir, 2018; Kazavchinsky et al., 2019) and pharmacological interactions between endogenous and exogenous molecules, such as between hormones and antidepressant drugs, between hormones and natural products, and between synthetic drugs and natural products (Estrada-Camarena et al., 2004, 2006; Shah et al., 2006; Poleszak et al., 2016; Cueto-Escobedo et al., 2020). Generally, antidepressant drugs reduce TTI in the FST in the absence of significant motor effects, which has been considered antidepressant activity (Koek et al., 2018; Unal and Canbeyli, 2019). Interestingly, a reduction of immobility that is produced by antidepressant drugs is associated with specific changes in serotonergic, noradrenergic, dopaminergic, and γ-aminobutyric acid-ergic neurotransmission (Shishkina et al., 2012; Ulloa et al., 2014), changes in neuronal activity (Contreras et al., 2000, 2001; Filho et al., 2015), and the expression of neurotrophic factors (Branchi et al., 2013; Filho et al., 2015; Vilela-Costa et al., 2021) in several brain structures that are involved in the neurobiology of depressive disorders.

Porsolt et al. (1977a) described in their original study that male Sprague-Dawley rats were forced to swim for 15 min in a Plexiglas cylinder (40 cm height, 18 cm diameter) that was filled with 25°C water. These rats exhibited long periods of immobility, mild hypothermia, and motor hypoactivity, which the authors considered “*a state of lowered mood in the rat*.” Interestingly, 24 h later, when the rats were forced to swim again for 5 min, they remained immobile for 75% of the test duration. This immobility was then reduced by antidepressant drugs (e.g., tricyclic antidepressants, monoamine oxidase inhibitors, and atypical antidepressants) and electroconvulsive shock, without significantly affecting motor activity. The authors considered that this reduction of immobility could be used as an effective tool that is “*capable of discovering new types of antidepressant agents hitherto undetectable using classical screening test*” (Porsolt et al., 1977a). The FST has also been adapted to evaluate the effect of antidepressant drugs in mice (Porsolt et al., 1977b; Yankelevitch-Yahav et al., 2015). Mice are forced to swim in a Plexiglas cylinder (24 cm height, 13 cm diameter) containing water (22 ± 2°C) to a depth of 10 cm. Different to rats, in mice there is only one session 6 min long divided into pretest (the first 2 min) and test (the last 4 min). Under these experimental conditions antidepressant drugs significantly also reduce TTI.

## Changes in Dimensions of Pools Used in the FST

The pool characteristics in Porsolt's original model (i.e., water depth, cylinder diameter, and water temperature) have been modified to evaluate the effect of antidepressant drugs on immobility in mice and rats (Borsini et al., 1986; Yates et al., 1991; Sunal et al., 1994). Although the influence of pool dimensions on immobility has been scarcely studied, evidence suggests that it may impact this behavior. Sunal et al. (1994) modified the diameter of the cylinder and water depth so that animals could touch the walls and bottom of the pool with their paws and tail. They used vertical cylinders with different diameters (10, 20, 30, and 50 cm) that were filled with water to a depth of 20 cm. The TTI was evaluated in male and female albino mice (Charles River) from 3 to 6 min in a single 15-min swim session. Under these conditions, higher immobility was detected when animals were tested in cylinders with the smaller diameters (10 and 20 cm), with an apparent influence of the mice being allowed to touch the walls with their paws. Conversely, lower immobility was detected in animals that were evaluated in cylinders with larger diameters (30 and 50 cm) because they could not touch the walls and thus were forced to swim (Sunal et al., 1994). Under these conditions, a single intraperitoneal (i.p.) injection of 10 and 20 mg/kg clomipramine and tranylcypromine, and 7.5 and 15 mg/kg maprotiline, significantly reduced TTI, but the pool dimension variable clearly affected TTI.

Other authors also modified the water depth from 15–18 to 30 cm (Detke and Lucki, 1996). Under this condition, male Sprague-Dawley rats exhibited high immobility and two additional behaviors, swimming and climbing. Swimming was associated with activation of the serotonergic system because selective serotonin reuptake inhibitors (20 mg/kg fluoxetine) increased swimming behavior, and climbing was related to activation of the noradrenergic systems because selective norepinephrine reuptake inhibitor (10 mg/kg desipramine) selectively increased this behavior after subcutaneous injection 23, 5, and 1 h prior to the test. Both antidepressant drugs significantly reduced TTI. Thus, modifications of pool characteristics permitted the identification of specific behaviors that are associated with the activation of particular neurotransmitter systems. Importantly, immobility behavior can be modified not only by antidepressant drugs but also by the dimensions of the pool that is used for the FST (Sunal et al., 1994). Methodological differences among laboratories can influence the measurement of immobility and subsequently identification of the potential antidepressant activity of drugs.

Contreras et al. (1995) introduced a rectangular pool (50 × 30 cm base, 60 cm height) to evaluate the effect of antidepressant drugs in Wistar rats. They evaluated TTI as an indicator of antidepressant-like activity and, additionally, measured the latency to the first period of immobility (LI). This latter variable was considered an indicator of the first effort of the rat to search for an exit and escape the stressful situation that was represented by the FST (Contreras et al., 1998). In this rectangular pool, Wistar rats injected with clomipramine (2.5 mg/kg/28-days, i.p.), desipramine (2.1 mg/kg/21-days i.p.), fluoxetine (0.5, 1, and 2 mg/kg/21-days i.p.), and Swiss albino mice treated with paroxetine (0.5, 1, and 2 mg/kg/21-days, p.o.) (Contreras et al., 1995, 1998, 2001; Amaghnouje et al., 2020a), as well as Wistar rats injected with progesterone (0.8, 1.6, and 3 mg/kg, 24 and 2 h before testing), allopregnanolone (1, 2, and 3 mg/kg, 1 h before testing) (Martinez-Mota et al., 1999; Molina et al., 1999a; Rodriguez-Landa et al., 2007; Cueto-Escobedo et al., 2020), some extracts (Wistar rats: 6.43 and 7.14 mg/kg/21-days *Hypericum perforatum* and 50 mg/kg/1–28-days *Montanoa frutescens* and *Montanoa grandiflora*; Swiss Albino mice: 250 and 500 mg/kg/1–21-days *Origanum majorana* extract, p.o.), and some metabolites from plants (Wistar rats: single doses of 1 mg/kg chrysin, i.p., Swiss albino mice: 50 and 100 mg/kg/1–21-days polyphenols, p.o.) (Lozano-Hernández et al., 2010; Rodríguez-Landa et al., 2018, 2020; Amaghnouje et al., 2020a,b) significantly reduced TTI and, some of them, increased LI. Therefore, the measurement of LI, in addition to TTI, may improve the detection of antidepressant-like activity in the FST (Castagné et al., 2009).

Other modifications of the dimensions of the FST pool have been reported. Using a small rectangular pool (30 × 26 cm base, 50 cm height) allowed the detection of a reduction of TTI that was associated with proestrus-estrus in female Wistar rats (i.e., a stage of the estrous cycle when steroid hormones are higher; Hernández-López et al., 2017). This same effect was detected when a single i.p. injection of 1 mg/kg chrysin in male Wistar rats was evaluated in this pool (Germán-Ponciano et al., 2020). One limitation of this small rectangular pool, however, was that LI was not influenced by chrysin, whereas this same doses of chrysin significantly reduced TTI and increased LI in a larger rectangular pool (Cueto-Escobedo et al., 2020; Germán-Ponciano et al., 2021). These findings indicate that pool dimensions can influence behavioral variables that are used to detect the potential antidepressant activity of drugs.

## Effects of Fluoxetine and Other Antidepressant Drugs on Immobility in the FST Using Cylindrical and Rectangular Pools

Generally, the doses of antidepressant drugs that are evaluated in a cylindrical pool are between 5 and 20 mg/kg (López-Rubalcava and Lucki, 2000; Fernández-Guasti et al., 2017; Abbasi-Maleki et al., 2020). Lower doses (e.g., <2 mg/kg) are generally ineffective in reducing immobility in mice and rats that are forced to swim (Detke et al., 1997; Estrada-Camarena et al., 2003: Vega-Rivera et al., 2016). Principally, fluoxetine is used as a pharmacological control for antidepressant activity to compare the effects of new substances with potential antidepressant activity in the FST. However, the effects of fluoxetine and other antidepressant drugs on immobility depend on dose, treatment duration, and pool characteristics (Table 1). The doses of fluoxetine that significantly reduce immobility in a cylindrical pool are > 5 mg/kg (López-Rubalcava and Lucki, 2000; Estrada-Camarena et al., 2003; Laino et al., 2014). In this pool, the acute administration (23.5 and 1 h before the FST) of fluoxetine and desipramine (1, 2, and 5 mg/kg, s.c.) in male Sprague-Dawley rats was devoid of effects on immobility, whereas 14 days treatment with 5 mg/kg fluoxetine and desipramine (Detke et al., 1997), significantly reduced immobility in a cylindrical pool. Similar effects were detected in ovariectomized Wistar rats with same treatment (Estrada-Camarena et al., 2003).

**Table 1:** Representative effects of fluoxetine and other antidepressant drugs on immobility behavior in the forced swim test using cylindrical and rectangular pools.

**Pool shape**	**Drug**	**Doses (mg/kg)**	**Treatment/method/ subject**	**Effect on immobility**	**Effect on latency to first episode of immobility**	**References**
Cylindrical	FLX	1, 2, and 5	a/nc/msdr	=	ne	Detke et al., 1997
		1 and 2	b/nc/msdr	=	ne	
		5	b/nc/msdr	↓	ne	
	FLX	5, 10, and 20	c/nc/msdr	↓	ne	López-Rubalcava and Lucki, 2000
	FLX	2.5 5 and 10	c/nc/ofwr c/nc/ofwr	= ↓	ne	Estrada-Camarena et al., 2003
	FLX	1 10	d/nc/mwr d/nc/mwr	= ↓	ne ne	Laino et al., 2014
	FLX	1.25	b/nc/ofwr	=	ne	Vega-Rivera et al., 2015
		10	b/nc/ofwr	↓	ne	
	FLX	5 and 10	e/nc/fwr	↓	ne	Fernández-Guasti et al., 2017
		5	e/nc/mwr	=	ne	
		10	e/nc/mwr	↓	ne	
	FLX	20	f/TIs/mnmrim	↓	ne	Abbasi-Maleki et al., 2020
	FLX	1 and 10	g/TIs/msm	↓	ne	Heinrich et al., 2021
	PRX	4 and 16	l/TIs/mm1	↓	ne	Leggio et al., 2008
	PRX	11	p/TIs/msm	↓	ne	Es-Safi et al., 2021
	CMI	5 and 10	l/TIs/mm1	↓	ne	Leggio et al., 2008
	CMI	15	m/TIs/msdr	↓	ne	García-Marquez et al., 1987
	CMI	1.25	n/TIs/mwr	=	ne	Takamori et al., 2001
	CMI	5	g/TIs/msdr	↓	ne	Liu et al., 2012
	IMI	10	f, o/TIs/msm	↓	ne	Citó et al., 2015
	IMI	5 and 10	n /TIs/mwr	↓	ne	Takamori et al., 2001
	IMI	30	l/TIs/mm2	↓	ne	Yan et al., 2015
	DMI	10, 20 and 30	g/TIs/mm1	↓	ne	Brielmaier et al., 2014
	DMI	6.25, 12.5 and 25	l/TIs/msm	↓	ne	Cassani et al., 2014
	DMI	10 and 30	l/TIs/msm	↓	ne	Yuen et al., 2017
Rectangular	FLX	0.5 1 and 2	g/TIs/mwr g/TIs/mwr	↓ ↓	= ↑	Contreras et al., 2001
	FLX	1	h/TIs/mwr	↓	↑	Lozano-Hernández et al., 2010
	FLX	1	h/TIs/mr	↓	↑	Rodríguez-Landa et al., 2018
	FLX	5	i/TIs/ofwr	↓	↑	Rodríguez-Landa et al., 2020
	FLX	1	j/TIs/mwr	↓	↑	Germán-Ponciano et al., 2021
	FLX	1	k/TIs/fwr	↓	=	Gutiérrez-García and Contreras, 2021
	FLX	1	g/TIs/fwr	↓	↑	Contreras et al., 2019
	PRX	11.5	p/TIs/msm	↓	ne	Amaghnouje et al., 2020a,b
	CMI	1.25	j/TIs/mwr	↓	ne	Molina et al., 1999a
	CMI	1.25	j/TIs/ofwr	↓	ne	Molina-Hernández and Téllez-Alcántara, 2001
	CMI	2.5	j/TIs/mwr	↓	ne	Molina-Hernández and Téllez-Alcántara, 2000
	IMI	2.5 and 5	b/TIs/mwr	↓	ne	Gutiérrez-García and Contreras, 2009
	IMI					Gutiérrez-García et al., 2007
	DMI	5	b/TIs/mwr	↓	ne	Contreras et al., 1998
	DMI	2.1	g/TIs/mwr	=	↑	Molina et al., 1999a
	DMI					Molina et al., 1999b
	DMI	2.14 32 10	j/TIs/mwr f/TIs/mwr n/TIs/mwr	↓ ↓ ↓	ne ne ne	Gutiérrez-García et al., 2003

*FLX, fluoxetine; PRX, paroxetine; CMI, clomipramine; IMI, imipramine; DMI, desipramine; a, 23.5 and 1 h before the FST; b, 14 days of treatment; c, 23.5, 5, and 1 h before the FST; d, 17 days of treatment; e, 24, 5, and 1 h before the FST; f, 45 min before the FST; g, 21 days of treatment; h, 28 days of treatment, but changes occurred beginning at 14 days of treatment; i, 24 and 2 h before the FST; j, 28 days of treatment; k, 5 h before the FST; l, 30 min before the FST; m, 13 days of treatment; n, 8 days of treatment; o, 15 days of treatment; p, 28 days of treatment, but changes occurred from day 1 of treatment; nc, number of counts; TIs, time of immobility in seconds; msdr, male Sprague-Dawley rats; ofwr, ovariectomized female Wistar rats; fwr, female Wistar rats; mwr, male Wistar rats; mnmrim, male NMRI mice; mm1, male C57BL/6j mice; msm, male Swiss mice; mm2, male C57BL/l mice; =, no effect; ne, not evaluated; ↓, reduction of immobility; ↑, increase immobility*.

Interestingly, chronic lower doses of fluoxetine (0.5, 1, and 2 mg/kg/21-days, p.o.) significantly reduced TTI when male Wistar rats were evaluated in a rectangular pool (50 × 30 cm base, 60 cm height), and 1 and 2 mg/kg fluoxetine also increased LI (Contreras et al., 2001, 2019). These doses were ineffective in the cylindrical pool. Long-term studies with a rectangular pool found a decrease in TTI and increase in LI that were produced by 1 mg/kg fluoxetine from day 7 to day 14 of treatment, and this effect persisted until days 21 and 28 of treatment (Lozano-Hernández et al., 2010; Rodríguez-Landa et al., 2018; Germán-Ponciano et al., 2021). The acute administration of 5 mg/kg fluoxetine 24 and 2 h before the FST significantly reduced TTI in a rectangular pool and increased LI (Rodríguez-Landa et al., 2020), a variable that was not evaluated in the cylindrical pool (Estrada-Camarena et al., 2003). These findings indicate that the pool dimensions in the FST and treatment schedules have an important influence on immobility behavior and other variables, such as LI, that can improve the detection of antidepressant-like activity of drugs.

## Concluding Remarks

Some modifications of pool dimensions in the FST (Sunal et al., 1994; Contreras et al., 2001), among other factors, have improved the identification of potential antidepressants drugs, but other modifications can also limit its detection (Germán-Ponciano et al., 2020). Few studies have evaluated the influence of pool dimensions on the effects of different doses of antidepressant drugs on immobility. An increase in the size or shape of the pool permits the measurement of other variables, such as the LI, in addition to the number of episodes of immobility and TTI that are generally evaluated in Porsolt's original model. Even a rectangular pool allows the identification of a decrease in TTI and an increase in LI with relatively low doses (0.5–2 mg/kg) of antidepressant drugs, such as fluoxetine, clomipramine and desipramine, which are otherwise ineffective in a cylindrical pool. This observation opens new directions to studying how changes in the dimensions of the pool that is used in the FST can contribute to more efficiently and effectively measuring immobility and other behaviors (e.g., swimming, climbing and diving, among others) as predictive of antidepressant activity in preclinical research. These observations should be considered when the FST is used to evaluate potential antidepressant activity of drugs.

## Author Contributions

JFR-L and GUR-S conceived the idea of the paper and developed its structure. GUR-S and LJG-P wrote the first draft of the manuscript. All authors selected and discussed the material to be included in the paper, reviewed, discussed, and approved the final version of the manuscript.

## Funding

GUR-S received a fellowship from Consejo Nacional de Ciencia y Tecnología (CONACyT-Mexico, Reg. 592165) for doctoral studies in Neuroethology. This research is part of the SIREI project (no. DGI: 266502021159) registered by JFR-L, with partial financial support from Sistema Nacional de Investigadores (SNI, Exp. 32753).

## Conflict of Interest

The authors declare that the research was conducted in the absence of any commercial or financial relationships that could be construed as a potential conflict of interest.

## Publisher's Note

All claims expressed in this article are solely those of the authors and do not necessarily represent those of their affiliated organizations, or those of the publisher, the editors and the reviewers. Any product that may be evaluated in this article, or claim that may be made by its manufacturer, is not guaranteed or endorsed by the publisher.
